# Our Daughters

**Published:** 1859-03

**Authors:** 


### OUR DAUGHTERS.

It is stated that in a female college at Harrodsburgh, Ky., the girls are taught to think, in the highest sense of the phrase. We hope President Reaser will establish a branch college of the same sort in New York city, there being nothing of the kind here. It seems that the "Euthalean Banner" is conducted by the young ladies connected with the institution. The name is puerile enough; better burn it up. It spoils both boys and girls to write for the papers, for it will not be done without diverting attention from study. A newspaper writer ought to have passed thirty years. The newspaper has become a too important institution to be written for by children; its responsibilities are becoming daily more momentous.

